# Differentiating between spinal schwannomas and meningiomas using MRI: A focus on cystic change

**DOI:** 10.1371/journal.pone.0233623

**Published:** 2020-05-29

**Authors:** Ji Hyun Lee, Hyun Su Kim, Young Cheol Yoon, Min Jae Cha, Sun-Ho Lee, Eun-Sang Kim

**Affiliations:** 1 Department of Radiology, Samsung Medical Center, Sungkyunkwan University School of Medicine, Seoul, Korea; 2 Department of Radiology, Chung-Ang University Hospital, Chung-Ang University College of Medicine, Seoul, Korea; 3 Department of Neurosurgery, Samsung Medical Center, Sungkyunkwan University School of Medicine, Seoul, Korea; George Washington University, UNITED STATES

## Abstract

**Objectives:**

To retrospectively determine the diagnostic ability of MRI in differentiating between intradural extramedullary spinal schwannomas and meningiomas.

**Methods:**

A total of 199 patients with spinal intradural extramedullary tumors who underwent preoperative contrast-enhanced MRI between January 2012 and December 2018 were included in this study. Two radiologists independently analyzed the presence of cystic change, dural tail sign, and neural foraminal extension. Clinical and MRI features between the two groups were compared by univariable and multivariable analyses using logistic regression. Interobserver agreements were calculated using kappa statistics.

**Results:**

Patients with schwannoma showed significantly higher frequency of cystic change (96% vs 24%, *P* < 0.001), neural foraminal extension (29% vs 3%, *P* = 0.001), and lumbar location (41% vs 5%, *P* = 0.008). Patients with meningioma showed significantly higher frequency of dural tail sign (64% vs 1%, *P* < 0.001), thoracic location (75% vs 31%, *P* = 0.007), older age (59.7 years vs 47.6 years, *P* < 0.001), higher female predominance (83% vs 50%, *P* < 0.001), and smaller size (19.8 cm vs 28.8 cm, *P* < 0.001). Multivariable analysis showed that cystic change (*P* < 0.001; odds ratio [OR], 0.02), dural tail sign (*P* < 0.001; OR, 36.23), age (*P* = 0.032; OR, 1.06), and lumbar location (*P* = 0.006; OR, 0.02) were independent factors. Interobserver agreements were almost perfect for all analyses.

**Conclusions:**

MRI features were useful in differentiating between intradural extramedullary schwannomas from meningiomas. The presence of cystic change and dural tail sign were independently significant discriminators.

## Introduction

Intradural extramedullary spinal tumors are the most common type of spinal tumors that may result in serious morbidity related to progressive neurologic deficits. [1–3] Among them, meningiomas and schwannomas are the two most common histologic subtypes, which comprise 55–90% of all intradural extramedullary spinal tumors. [4,5] Although both tumors are mostly benign, there is a substantial difference between the surgical techniques for removing each. [6,7] Since spinal meningiomas have a tendency to recur, complete tumor resection with dural excision is recommended whenever practicable to lower the recurrence rate. [6,8,9] Thus, radiologic differentiation between these two spinal tumors is a crucial step in surgical planning.

Magnetic resonance imaging (MRI) serves a critical role in evaluating patients suspected to have spinal tumors by detecting and characterizing the lesions. [10–12] Many studies have reported MRI to be useful in differentiating between intradural extramedullary schwannomas and meningiomas. [1,7,13,14] Besides locating the tumor, dural tail sign or neural foraminal extension was suggested as a useful imaging feature in differentiating between the two histologic subtypes. [4,7,13,14] Signal intensity of the tumor on T2-weighted image was also shown to be helpful for the differentiation; schwannomas have a tendency to show a hyperintense signal while a substantial portion of meningiomas shows an isointense signal compared to that of the spinal cord. [7,13,14] However, most of the reported studies involve few cases, and the discriminating ability of each sign varies among studies. [1,4,7,13,14] Thus, there is room for further investigation to identify other and better imaging features that could help in easily differentiating between subtypes of intradural extramedullary spinal tumors.

Schwannomas are histologically characterized by the presence of alternating areas with two distinct spindle cell arrangements; Antoni A region, a highly cellular area, and Antoni B region, which is a loosely organized myxoid tissue. [15,16] Cells within the Antoni B regions are often separated from one another by microcysts filled with mucin. [17] It was suggested that these microcysts could coalesce to form macrocyst, which can be detected on MRI. [15,18] In addition, vascularization of Antoni B region is reported to contain degenerative features, and further degeneration in this region can form larger cyst. [15] A heterogeneous high signal with immeasurably small foci of fluid signal within the tumor demonstrated on a T2-weighted image was reported to represent histopathological cyst formation. [14,19] We assumed that the heterogeneous high T2 signal and large cyst formation within the tumor are part of a continuum with different levels of degeneration, which may represent histological microcyst and macrocyst formation, respectively. Thus, we used the term “cystic change” to describe a single imaging feature that encompasses this spectrum of findings. This imaging finding has not been widely studied in depth as a single imaging parameter.

We hypothesized that the presence of cystic change on T2-weighted image can help in differentiating between intradural extramedullary schwannomas and meningiomas. Therefore, we performed a retrospective study on MRI features for differentiating between intradural extramedullary schwannomas and meningiomas, with a special focus on the diagnostic performance of cystic change as demonstrated on T2-weighted images.

## Methods and materials

### Study subjects

This retrospective study was approved by the institutional review board (BLINDED), which waived the requirement to obtain informed consent. We initially identified 231 consecutive patients who underwent surgical removal of spinal intradural extramedullary tumors at our institution, and had preoperative contrast-enhanced spine MRI between January 2012 and December 2018.

Intradural extramedullary tumors other than schwannomas and meningiomas were excluded because they are not directly relevant to this study. The included 27 tumors are as follows: myxopapillary ependymomas (n = 9), lipomas (n = 3), neurofibromas (n = 3), melanocytomas (n = 3), paragangliomas (n = 2), angiolipoma (n = 1), inflammatory pseudotumor (n = 1), pilocytic astrocytoma (n = 1), dermoid cyst (n = 1), neurenteric cyst (n = 1), lymphoma (n = 1), and metastasis (n = 1).

The overall image quality defined by several factors including motion artifact and diagnostic acceptability for tumor evaluation was evaluated by two radiologists in consensus. A 4-point scale was used: 1, very poor image quality; 2, suboptimal image quality; 3, mild artifacts not affecting image evaluation; and 4, optimal. MR images with grade 3 and 4 were considered acceptable. Images of 5 patients (2 schwannomas and 3 meningiomas) were rated as grade 2, and were excluded.

**Fig 1:**
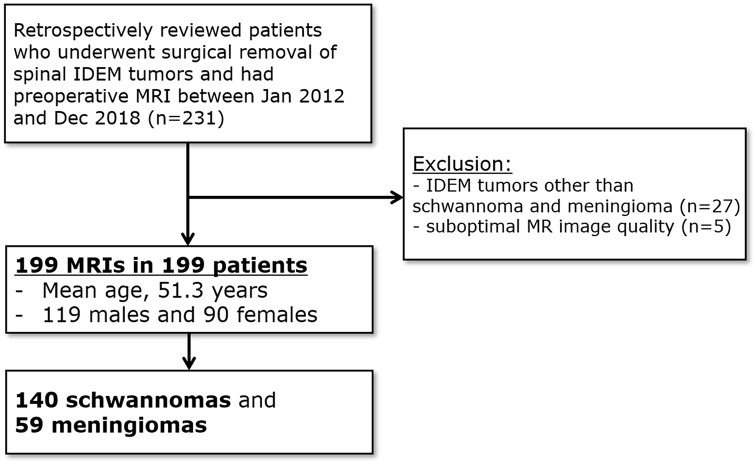
Patient flow diagram. IDEM, intradural extramedullary.

Finally, 199 MR images of 199 patients (119 women, 80 men; age range 7–84 years; mean 51.3 years), including 140 patients with schwannoma and 59 patients with meningioma, were included in this study (Fig 1).

### MRI acquisition

Among the 199 MR images, 106 were obtained at our institution and 93 were obtained at other institutions before referral to our hospital. MRI equipment and protocols (e.g., field of view, matrix size, slice thickness, echo train length, etc.) varied according to the institution, as well as the anatomical position that the imaging was performed in. Most of the scans performed in our institution used either a 3-T system (Intera Achieva or Ingenia, Philips Medical Systems, Best, The Netherlands) or a 1.5-T system (Signa CV/I; General Electric Medical System, Milwaukee, WI). Standard MRI protocol of our institution comprise sagittal turbo spin-echo T1-weighted image (repetition time [TR]/ echo time [TE], 422–875 ms/ 9–23 ms), sagittal and axial T2-weighted images (TR/TE, 3,000–4,500 ms/ 96–120 ms), sagittal and axial T1-weighted images with or without fat suppression, after intravenous administration of gadoterate meglumine (Gd-DOTA, Dotarem^®^, Guerbet, Roissy CdG Cedex, France). All the MR images obtained at other institutions also included T2-weighted images and post-contrast T1-weighted images in the sagittal and axial planes.

### Clinical and imaging parameter analysis

Clinical data, including age, sex, tumor histopathology, date of MRI examination and surgery were gathered, through reviewing of electronic medical records.

Two board-certified radiologists (BLINDED and BLINDED, with 3 and 5 years of experience in musculoskeletal MRI, respectively) blinded to clinical information and histopathologic results independently evaluated the MR images using a picture archiving and communication system (Centricity Radiology RA 1000; GE Healthcare, Chicago, IL, USA) without data anonymization. Each radiologist evaluated the presence of cystic change, dural tail sign, and neural foraminal extension. Cystic change was considered present when heterogeneously high signal intensity with immeasurably small fluid signal or measurable cyst formation was seen within tumor on T2-weighted image, using cerebrospinal fluid as a reference. Dural tail sign was considered positive if thickening with enhanced dura mater adjacent to the tumor that taper away from it was demonstrated on the contrast-enhanced image. [14,20] Neural foraminal extension was considered present if the tumor occupying the neural foramen on axial or sagittal T2-weighted image was seen regardless of the presence of neural foraminal widening or bone erosion.

One radiologist with 5 years of experience in musculoskeletal MRI (BLINDED), recorded the location (cervical, thoracic, lumbar, and sacral region) of each lesion and size of the tumor. Location of the tumors involving the cervicothoracic, thoracolumbar, or lumbosacral junction were defined as the region affected by more than half the volume of the tumor. The size of the tumor was defined as its largest dimension among the lesion’s longitudinal, anteroposterior, and transverse dimensions measured on a T2-weighted image.

### Statistical analysis

Continuous and categorical variables were summarized as means with standard deviations and frequency (%), respectively. Logistic regression analysis was performed to assess the difference in clinical and MRI parameters between schwannoma and meningioma for univariable and multivariable analysis using Firth’s correction. The goodness-of-fit was checked using the Hosmer-Lemeshow test.

The area under the curve (AUC) was calculated based on a receiver-operating characteristic curve analysis for cystic change, dural tail sign, neural foraminal extension, and combination of significant parameters based on the logistic regression model; differences between the AUCs were assessed according to DeLong et al.’s method. [21] The optimal cutoff to distinguish meningioma from schwannoma was determined by maximizing Youden’s index, and the sensitivity, specificity, accuracy, positive and negative predictive values were calculated.

Kappa statistics was used to calculate the interobserver agreement between the readers, regarding cystic change, dural tail sign, and neural foraminal extension. The degree of agreement was interpreted as ‘poor’ for a κ value of less than 0, ‘slight’ for a κ value of 0–0.20, ‘fair’ for a κ value of 0.21–0.40, ‘moderate’ for a κ value of 0.41–0.60, ‘substantial’ for a κ value of 0.61–0.80, and ‘almost perfect’ for a κ value of 0.81–1.0. All statistical analyses were performed using Statistical Analysis Software (version 9.4, SAS Institute, Cary, NC, USA) and MedCalc version 18.11.3 (MedCalc Software bvba, Ostend, Belgium; http://www.medcalc.org; 2019). *P* < 0.05 was considered statistically significant.

## Results

Among the 199 tumors, 48 tumors (36 schwannomas and 12 meningiomas) were located in the cervical spine, 88 (44 schwannomas and 44 meningiomas) in the thoracic spine, 60 (57 schwannomas and 3 meningiomas) in the lumbar spine, and 3 (schwannomas) in the sacral spine. The mean and median size of the tumors were 2.61 cm and 2.10 cm (range, 0.60–15.20 cm), respectively. The average interval between MRI examination and surgery was 29.42 days (range, 0–136 days).

**Fig 2:**
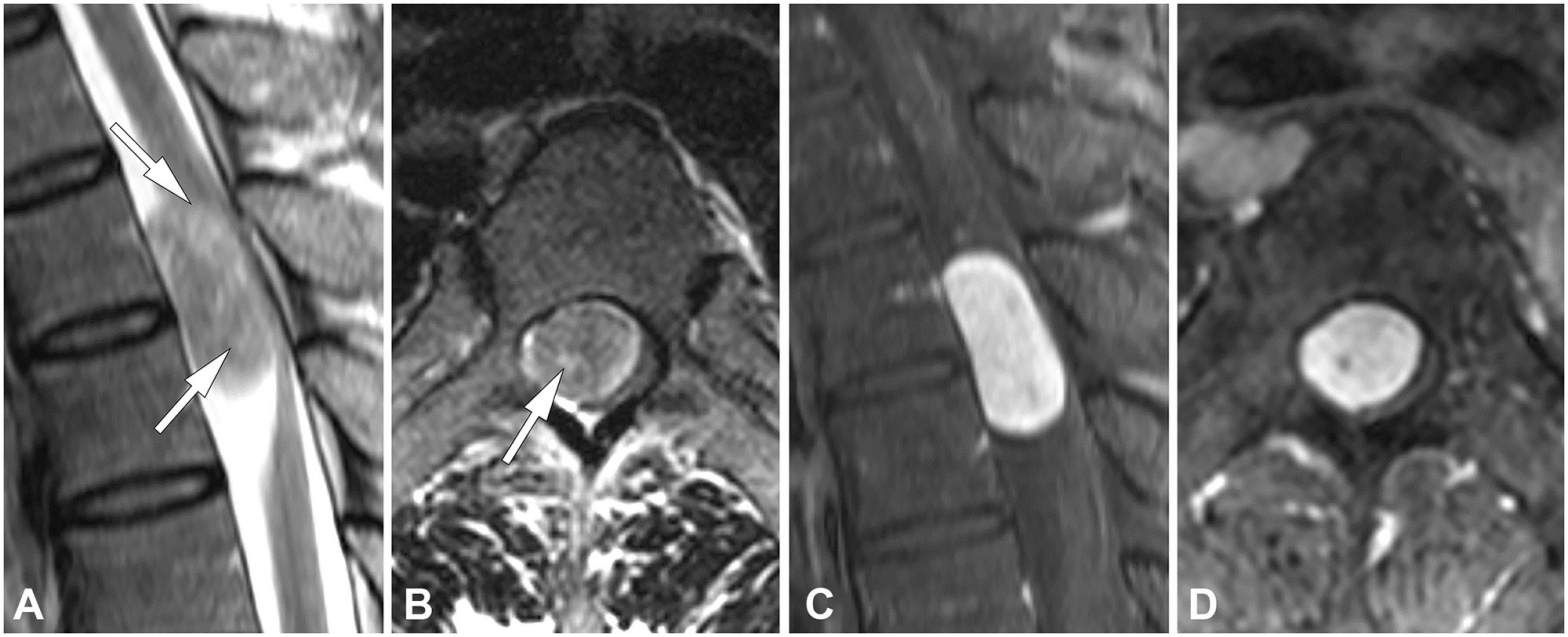
A schwannoma in a 42-year-old man. (**A**) Sagittal and (**B**) axial T2-weighted images show an intradural extramedullary mass on the T4-5 level with immeasurably small foci of fluid signal within the tumor (arrows), which was considered to represent cystic change. (**C**) Sagittal and (**D**) axial fat-suppressed contrast-enhanced T1-weighted images show relatively homogeneous enhancement.

**Fig 3:**
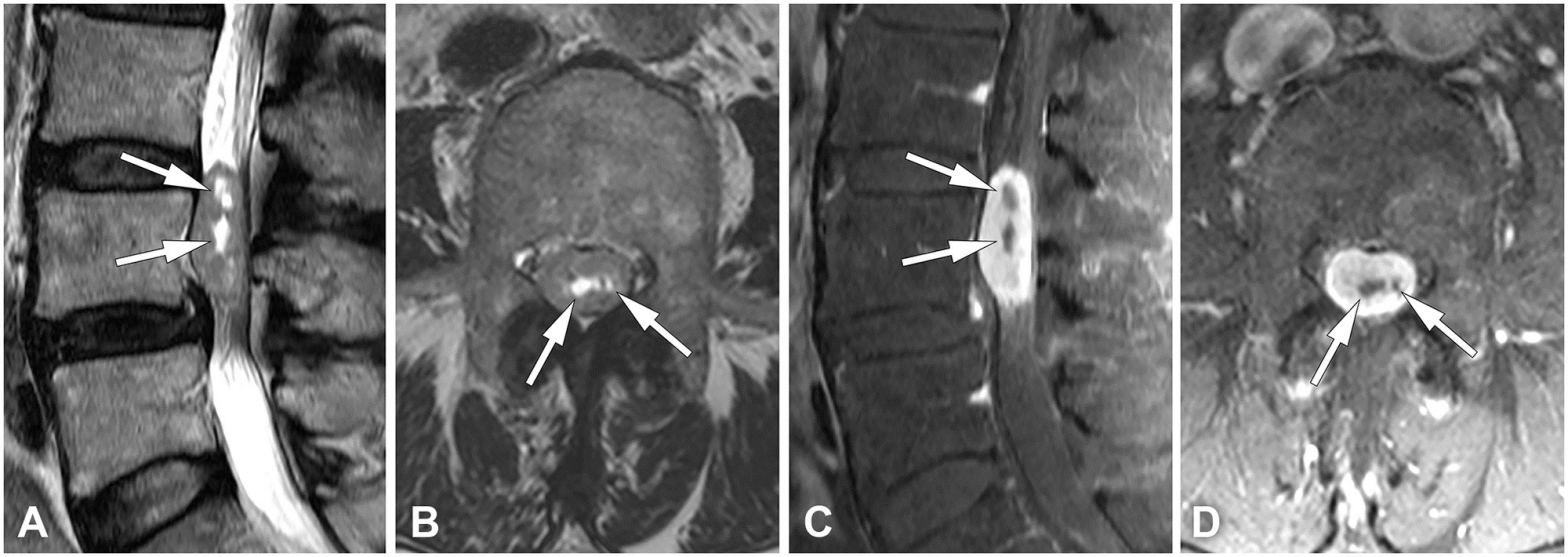
A schwannoma in a 59-year-old woman. (**A**) Sagittal and (**B**) axial T2-weighted images show an intradural extramedullary mass on the L4 level with measurable cyst formation (arrows). (**C**) Sagittal and (**D**) axial fat-suppressed contrast-enhanced T1-weighted images show relatively homogeneous enhancement except for non-enhancing cystic portions (arrows).

**Fig 4:**
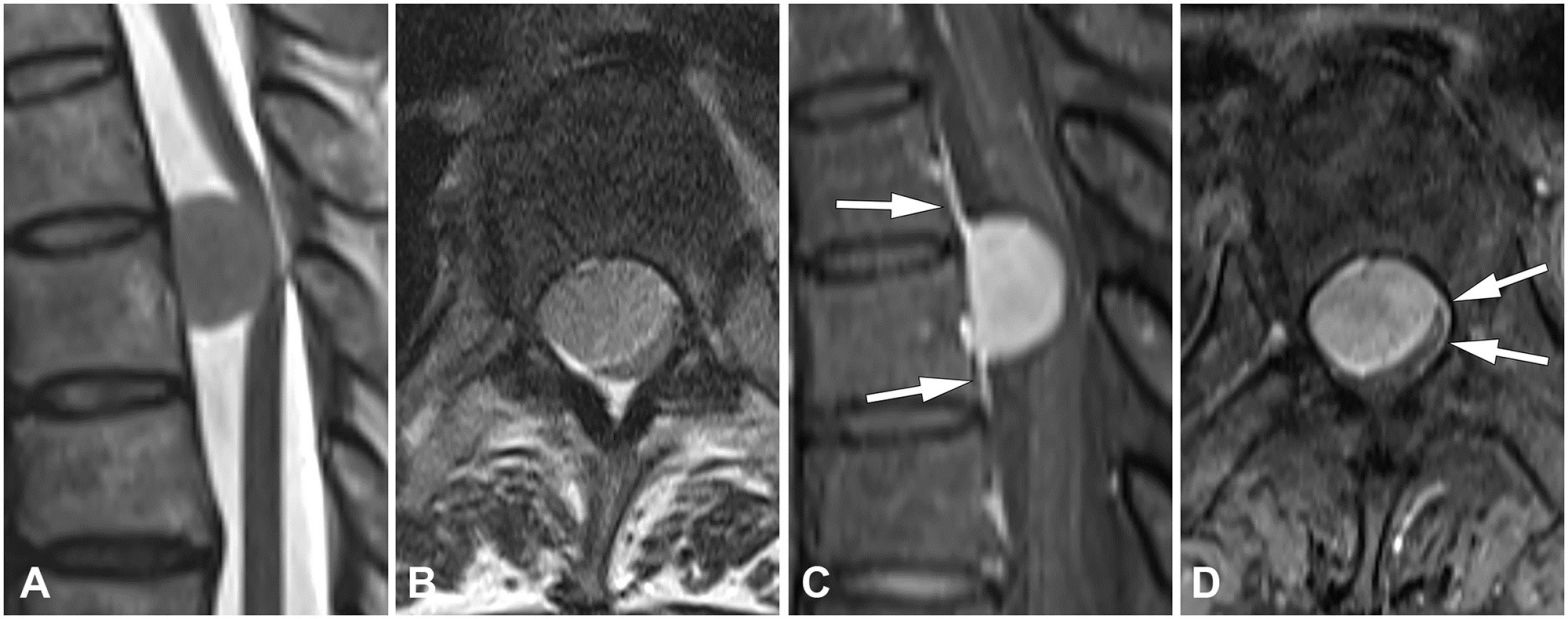
A meningioma in a 53-year-old woman. (**A**) Sagittal and (**B**) axial T2-weighted images show an intradural extramedullary mass on the T12 level that is slightly hyperintense than the spinal cord (**C**) Sagittal and (**D**) axial fat-suppressed contrast-enhanced T1-weighted images show thickening with enhancement of dura mater adjacent to the tumor that tapers away from it, representing dural tail sign (arrows).

The statistical significance of clinical and MRI parameters for schwannoma and meningioma on univariable and multivariable analyses are summarized in Table 1. All the characteristics were significantly different between the two groups in the univariable analysis. Patients with schwannoma showed significantly higher frequency of cystic change, neural foraminal extension, and lumbar location (Figs 2 and 3). Patients with meningioma showed significantly higher frequency of dural tail sign, thoracic location, older age, higher female predominance, and smaller size (Fig 4). Multivariable analysis showed that cystic change, dural tail sign, age, and lumbar location were independent factors. Chi-square and *P* values from the Hosmer–Lemeshow test were 4.30 and 0.829, respectively.

**Table 1:** Comparison of clinical and MRI parameters in differentiating between schwannoma and meningioma groups.

	Univariable analysis			Multivariable analysis	
	Schwannoma	Meningioma	*P*	OR (95% CI)	*P*
**All tumors (n = 199)**					
**Age (years)** ^†^	47.6 ± 14.5	59.7 ± 12.0	< 0.001	1.06 (1.01–1.11)	0.032
**Gender (male)**	70 (50%)	10 (17%)	< 0.001	0.30 (0.06–1.41)	0.127
**Size (cm)** ^†^	28.8 ± 16.8	19.8 ± 10.6	< 0.001	0.95 (0.89–1.01)	0.076
**Location**					
**Cervical**	36 (26%)	12 (20%)	ref	N/A	N/A
**Thoracic**	44 (31%)	44 (75%)	0.007	0.69 (0.15–3.24)	0.638
**Lumbar**	57 (41%)	3 (5%)	0.008	0.02 (< 0.01–0.33)	0.006
**Sacral**	3 (2%)	0 (0%)	0.623	< 0.01 (< 0.01->999)	0.987
**Cystic change (%)**	134 (96%)	14 (24%)	< 0.001	0.02 (< 0.01–0.12)	< 0.001
**Dural tail sign (%)**	2 (1%)	38 (64%)	< 0.001	36.23 (5.03–261.26)	< 0.001
**NF extension (%)**	40 (29%)	2 (3%)	0.001	1.21 (0.17–8.59)	0.849

^†^ Data are means with standard deviations.

NF indicates neural foraminal; OR, odds ratio; CI, confidence interval; N/A, not applicable; and ref,

The equation for multiple logistic regression analysis was constructed as the following Eq (A), whereas *n* represents 0, 0.3709, 3.9017, and 11.8205 for cervical, thoracic, lumbar and sacral location, respectively:

y=0.5749+0.0545×age(years)−0.0564×size(mm)−1.2125(ifmale)−n−3.7527(ifcysticchangepresent)+3.5899(iftailsignpresent)+0.19(ifneuralforaminalextensionpresent) (A)

The probability (*p*) of meningioma for each patient could be calculated by inserting *y* into the Eq (B), providing a probability value between 0 and 1:

$$p=\frac{e^{y}}{{1+e}^{y}}$$ (B)

The overall diagnostic performances of MRI parameters for differentiating two groups are shown in Table 2. Among single parameters, cystic change showed the highest AUC (0.860, 95% CI 0.804–0.905), which was significantly higher than that of neural foraminal extension (*P* < 0.001). However, there was no significant difference (*P* = 0.234) between AUCs of cystic change and dural tail sign. Combination of significant parameters showed the highest AUC (0.975, 95% CI 0.943–0.992) that was significantly higher than all the single parameters (*P* < 0.001).

**Table 2:** Diagnostic performances of the MRI parameters.

	Sensitivity	Specificity	Accuracy	PPV	NPV	AUC
**All tumors (n = 199)**						
**Cystic change**	76.3%	95.7%	86.0%	88.2%	90.5%	0.860 (0.804–0.905)
**Dural tail sign**	64.4%	98.6%	81.5%	95.0%	86.8%	0.815 (0.754–0.866)
**NF extension**	96.6%	28.6%	62.6%	36.3%	95.2%	0.626 (0.555–0.693)
Combination*	89.8%	97.1%	93.5%	91.4%	95.7%	0.975 (0.943–0.992)

* Based on the logistic regression Eq (A). Sensitivity, specificity, accuracy, PPV, and NPV values were calculated using cutoff 0.3766 that maximized Youden’s index.

Numbers in parentheses are 95% confidence intervals.

AUC indicates area under the curve; PPV, positive predictive value; NPV, negative predictive value; and NF, neural foraminal.

Interobserver agreements were almost perfect for all parameters (cystic change κ = 0.921, 95% confidence interval [CI]: 0.859–0.983; dural tail sign κ = 0.969, 95% CI: 0.926–1.000; neural foraminal extension κ = 1.000, 95% CI: 1.000–1.000), and data obtained by one of the readers were used for comparison.

## Discussion

The goal of our study was to investigate whether MRI features can help differentiation of intradural extramedullary schwannomas and meningiomas, specially focusing on the cystic change of tumors on T2-weighted image. This study included a relatively large number of cases, and the results showed that MRI features, which include cystic change, can be used as a useful imaging marker for differentiating between intradural extramedullary schwannomas and meningiomas. Constructed model based on multiple logistic regression using clinical and MRI parameters including cystic change provided excellent diagnostic performance for discriminating the two.

To our knowledge, only a few studies have reported on the histologic correlation of MRI features of spinal schwannomas. The exact mechanism of histologic cyst formation within schwannoma is unknown, but it has been speculated that the coalescence of mucinous or microcystic areas in Antoni B areas may result in cyst formation. [15,22,23] In a study on vestibular schwannomas, larger tumors were reported to have a tendency to demonstrate increased T2 signal intensity, higher portion of Antoni type B tissue, and microscopic cyst formation. [18] Whereas cystic meningioma is reported to comprise 3.5% of intracranial meningiomas, [24,25] cystic change in spinal meningioma is reported to be exceedingly rare, with only few case reports in the literature. [26,27] On the assumption that cystic change can be used to differentiate schwannomas from meningiomas, we investigated the differentiating ability of cystic change on T2-weighted images and obtained promising results; cystic change turned out to be a significant discriminator of the two tumor groups, presenting a significantly higher frequency in schwannoma in both univariable and multivariable analyses.

Previous studies suggested that signal intensity of intradural extramedullary spinal tumor on T2-weighted image is a potential discriminator of schwannoma and meningioma. [4,13,14] However, not much attention has been centered on cystic change and its evaluation as a single imaging parameter. Furthermore, previous studies used inconsistent definitions and terminologies, and lack precise explanations on the size of their findings within tumors with most of the reported studies on radiologic differentiation including limited number of cases. Although Verdelhan et al. [14] analyzed T2 signal characteristics to notice that of schwannoma was significantly hyperintense and heterogeneous, the presence or absence of cystic change was not determined. Takashima et al. [7] reported that the ratio between T2 signal intensity of tumor and that of subcutaneous fat was significantly higher in spinal schwannomas compared to that of meningiomas. In terms of T2 signal intensity, our results were partially comparable to these studies. Liu et al. [13] reported the largest series to date and further categorized T2 signal of tumors with addition of fluid signal intensity. Iwata et al. [4] and Zhai et al. [28] analyzed the presence of cystic degeneration defined as signal intensity equal to that of the cerebrospinal fluid exhibiting low intensity on T1-weighted image and high intensity on T2-weighted image, which turned out to be a useful parameter for predicting schwannoma. However, they also did not provide clear definition on the size of the fluid signal or cystic change containing lesions within the tumor. On the other hand, our study defined the term “cystic change” to encompass both microcyst and macrocyst to demonstrate its usefulness for differentiation between spinal schwannoma and meningioma in a large patient population.

In accordance with previous reports, dural tail sign and neural foraminal extension was significantly common in meningiomas and schwannomas, respectively when compared with their counterpart. [1,4,13,14] Meningioma with neural foraminal extension, also called dumbbell-type meningioma, is considered relatively rare. [6,26,29] In our study, only 2 cases (3%) of meningioma showed neural foraminal extension, which was comparable with the previous reports. Dural tail sign was also an independent factor in multivariable analysis to differentiate schwannomas from meningiomas.

In addition, gender, age, and tumor location were reported to differ between spinal schwannoma and meningioma in previous literatures. [13,30–32] Schwannomas and meningiomas were preferentially located in the lumbar and thoracic regions, respectively, and meningiomas showed older age and female predominance. Although no direct comparison was conducted, intradural extramedullary meningiomas tended to be smaller than schwannomas. [13] Our result is in accord with these previous studies.

In addition to the intrinsic limits of a retrospective study, there were several limitations in our study. First, substantial number of MR images was obtained using different MRI scanners and protocols at a number of clinics before referral to our institution. Differences in MRI parameters and contrast enhancement protocols may have affected our decision on the presence of intratumoral cystic change and dural tail sign. Second, only schwannomas and meningiomas were considered, excluding other rarer intradural extramedullary tumors. Furthermore, we did not perform histologic correlation for the assessment of cystic change in tumors. Fourth, the presence of cystic change was determined based on subjective analysis. Although the high interobserver agreement might have justified this method, it would be more desirable to have a quantitative measure that can define the presence of cystic change for better reproducibility. Future studies using texture analysis may provide the appropriate methodology regarding this issue. Lastly, findings on computed tomography such as intratumoral calcification were not sought.

## Conclusion

In conclusion, our results suggest that MRI can provide useful data to differentiate between intradural extramedullary schwannomas and meningiomas. The presence of cystic change and dural tail sign was an important discriminator of the two histologic subtypes. Further investigations on histologic correlation are needed to validate our results.

## Supporting information

S1 Data (XLSX)
